# In Situ Cranioplasty for Renal Cell Skull Metastasis: Technical Note

**DOI:** 10.7759/cureus.4128

**Published:** 2019-02-24

**Authors:** John F Burke, Vivek Sudhakar, Steve Braunstein, Michael McDermott

**Affiliations:** 1 Neurosurgery, University of California - San Francisco, San Francisco, USA; 2 Radiation Oncology, University of California - San Francisco, San Francisco, USA

**Keywords:** cranioplasty

## Abstract

Treatment of a large, symptomatic skull metastasis requires surgical excision and in many cases postoperative radiation therapy. Immediate reconstruction of the skull for cerebral protection usually involves cranioplasty with titanium mesh and/or methyl methacrylate. Preoperative synthetic cranioplasty technology is yet to evolve sufficiently to allow computer-generated prostheses to precisely fit a defined craniectomy defect created at the time of tumor removal. We document the techniques used for simultaneous craniectomy and composite cranioplasty in the setting of a large occipital renal cell skull metastasis. Preoperative computed tomography (CT) and magnetic resonance (MR) imaging identified the pathological anatomy of an occipital skull metastasis presenting as an exophytic scalp mass. Preoperative angiography and embolization was performed followed by craniectomy in the semi-sitting position and composite cranioplasty using titanium mesh and methyl methacrylate. A series of steps in the surgical procedure are outlined to assist with safely and accurately performing the craniectomy and cranioplasty to guarantee the best surgical and cosmetic outcome. Postoperative CT imaging confirmed excellent contours of the cranioplasty. The method described herein allows for a single-step surgical procedure to excise a large skull metastasis and create a structurally sound and cosmetically acceptable composite cranioplasty. This method can also be used for the excision and repair of other skull tumors or anomalies requiring excision.

## Introduction

Patients with early cranioplasties have been reported to demonstrate improvements in neurological function. These improvements are posited to be due to restoration of normal cerebral spinal fluid (CSF) flow dynamics and a resolution of a phenomenon termed the “syndrome of the trephined” [1-3]. Currently, the existing skull defects are routinely repaired with computer-generated cranioplasty implants. A variety of materials are used to construct these implants, including methyl methacrylate, polyether ether ketone (PEEK) [4], and other polymers.

The excision of large, symptomatic skull lesions, such as primary or secondary skull neoplasms, often requires a simultaneous cranioplasty. However, excising a large, symptomatic skull lesion and creating a simultaneous cranioplasty with an appropriate shape and contour is problematic for surgeons. It is difficult to repair a large skull defect immediately following the excision of a pathological lesion. In particular, repairs are complicated by the need to maintain the tensile and compressive strength of the skull while also ensuring a cosmetic/symmetric result. This is especially important in nonhair bearing scalp locations.

Bloch and McDermott previously described a so-called “in situ” cranioplasty for the excision and reconstruction of cranial vault hyperostosing meningiomas [5]. The same technique is applicable to other lesions of the skull such as metastatic tumors and fibrous dysplasias. Performing a simultaneous repair avoids the need for a second surgical procedure to implant a computer-generated prosthesis after a period of risk exposure from a large skull defect.

We describe the technique of in situ cranioplasty used for the excision of a large occipital renal cell skull metastasis and point out the sequence of steps that may be used to ensure a safe excision and structurally sound and cosmetically acceptable cranioplasty performed at the time of tumor removal.

## Technical report

**Figure 1:**
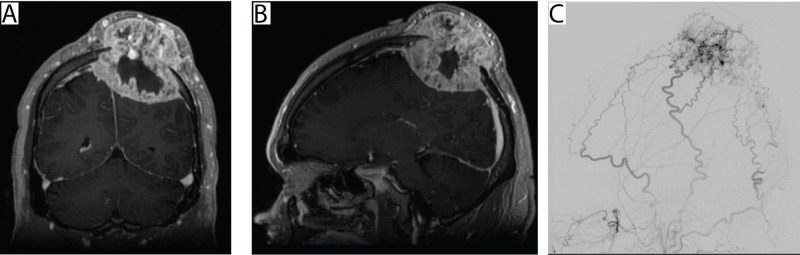
Preoperative imaging. A: Postcontrast T1 weighted coronal magnetic resonance (MR) image showing a calvarial-based metastatic lesion invading the calvarium over the left parietal-occipital region. B: Postcontrast T1 weighted sagittal MR image showing the same lesion. C: Preoperative angiogram showed that the tumor had arterial supply from multiple branches of the bilateral superficial temporal, middle meningeal, and occipital arteries as well as intracranially from the left anterior falcine artery.

A 43-year-old man presented with a large occipital scalp mass that had been growing quickly over the prior three months. His local oncologist had performed a body positron emission tomography (PET) CT revealing a hypermetabolic kidney mass consistent with renal cell carcinoma, as well as a large occipital skull mass. Figure 1 shows the preoperative MRI of the skull lesion in panels A and B. The patient was referred for surgical excision and cranioplasty preceded by angiography and embolization; preoperative angiogram is shown in Figure 1C. Image guided volumetric MRI images were obtained with fiducials for intra-operative image guidance.

**Figure 2:**
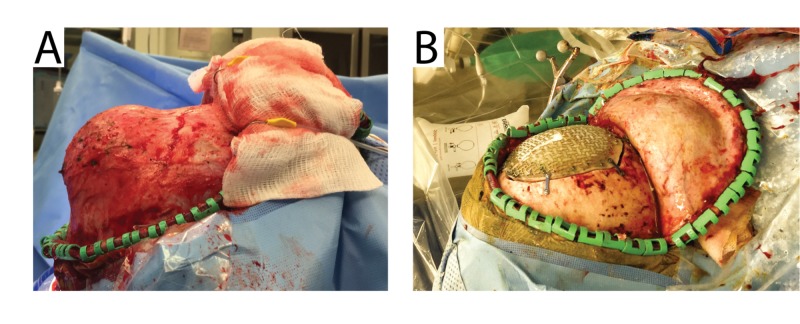
Intraoperative photographs. Photographs show the tumor both before (A) and after (B) resection and in situ cranioplasty. The titanium mesh is seen attached to the 1.5 cm strip of native bone around the original bony tumor site. The tumor was resected and the resulting bony defect was replaced with composite cranioplasty material.

The patient was positioned semi-sitting with the skull clamp positioned anteriorly and connected to a table attachment over the body of the patient (Figure 2). Preoperative bubble echocardiography had been done to confirm there was no open atrial septal defect. Precordial ultrasound monitoring and central venous access was included to detect and treat any significant intraoperative venous air embolism.

Following registration of imaging to physical space, the margin of tumor-involved bone under the scalp was outlined to assist with scalp incision planning. A bicoronal-parietal-occipital incision was marked extending from the vertex down to behind both ears. The scalp was opened, and the subgaleal plane was dissected using electrocautery for hemostasis. As the pericranium was removed from over the tumor-involved skull, bleeding was controlled with both absorbable and nonabsorbable bone wax. The margin of the tumor in bone was then outlined on the skull and a separate margin of 1.5 cm of additional bone in a ring around this. The protruding skull with tumor was then drilled down using a 6 mm cutting burr. Enough bone was removed to blend in with the contours of the surrounding skull and to recreate the occipital curvature when viewed in profile from either side. A large piece of titanium mesh was then molded and cut to the shape of the drilled down tumor-involved skull. The molded mesh was then placed on the back table. 

Next the 6 mm cutting burr was used to drill a trough in the bone around the skull metastasis down to a thin shell over the dura. Hemostasis was maintained using malleable absorbable bone wax. Once the circumference of the tumor had been drilled the remaining thin layer of bone at the depth of the trough was removed with a 3 mm Kerrison after dissecting the dura away from the bone. The isolated central portion of the skull invaded by tumor, which was very adherent to the underlying dura, was removed in pieces using the footplate attachment of the drill and Leksell rongeurs. The surrounding 1.5 cm rim of normal skull was then removed in a single circular piece and the dura tacked up at the edge of the craniectomy in standard fashion.

As the tumor did not penetrate the dura, soft tissue in the epidural space was scraped off using the Penfield #1 and the dura was coagulated throughout. The previously molded mesh was then taken from the side table and was screwed into the surrounding circular bone flap using 4 mm screws. The inner circumference of the circular craniectomy was traced with a surgical marking pen on a paper template. We then used this template to estimate the size of the methyl methacrylate piece for the composite construct. The plastic was mixed and, after a period of time to allow for setting of the polymer, it was then cut to the approximate shape of the paper template. Then, the cut polymer was placed into the inner table defect and secured onto the interstices of the outer table mesh. Excess plastic was scrapped off the outer table side and the material was allowed to harden off the field where the exothermic event would cause no tissue damage.

**Figure 3:**
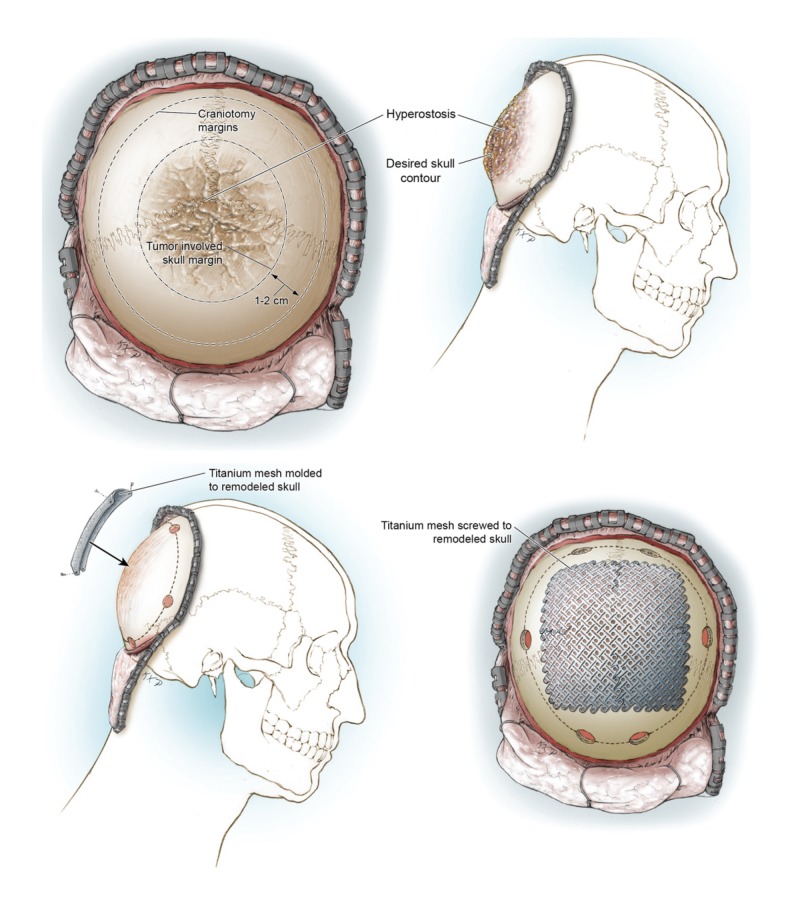
Illustrations of the in situ cranioplasty method. Top panel: the bone is divided into three sections: (1) tumor-involved bone; (2) native bone in a rim 1-2 cm around the tumor-involved bone (1.5 cm was used in the case report). This bone will be removed for the cranioplasty; (3) native bone not involved with the cranioplasty. Bottom panel: the titanium mesh is fit to the tumor-involved bone, which is subsequently resected. Illustrations by Ken Probst

The composite cranioplasty was then secured to the surrounding skull with titanium plates and screws, irrigated with 3% betadine solution, then saline, followed by the application of Vancomycin powder. A 1/8” hemovac drain was placed in the subgaleal space and the scalp closed in the standard fashion. The entire procedure is summarized in Figure 3.

**Figure 4:**
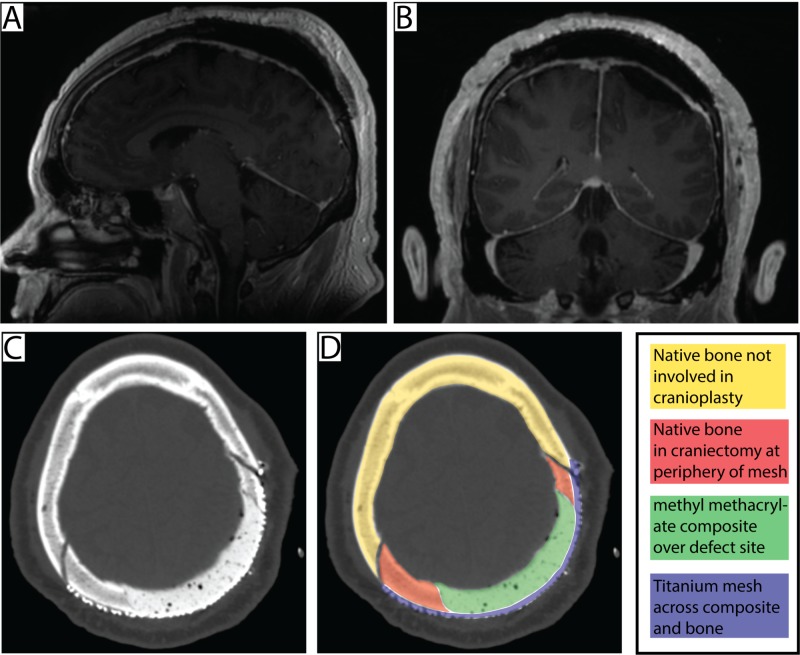
Postoperative imaging. A: Sagittal and B: coronal postcontrast T1 weighted postoperative MR image showing gross total resection of the metastatic lesion. C: bone weighted postoperative CT scan showing the cranioplasty site. D: The different areas of bone involved in the cranioplasty are shown color coded based on the image in C.

The patient’s postoperative course was uncomplicated. Postoperative MR and CT imaging revealed an appropriate contour of the cranioplasty and gross total tumor excision (Figure 4). The patient was recommended for external beam radiotherapy. Upon discharge he has been doing well and has had no complaints regarding his excision site, no wound infection, and no issues with the healing process. At one-year follow up, he was doing well and surveillance MR imaging revealed no tumor recurrence. 

## Discussion

Metastatic lesions to the central nervous system can either be resected, irradiated, or treated with both surgery and radiation. Increasing data have shown that, for larger lesions, surgery and radiation offer the best source control for metastatic lesions. When lesions are completely intra-axial, surgical options can be more straightforward and usually involve a typical craniotomy for tumor excision, much like a primary glioma. However, lesions that primarily involve the calvarium also need to be resected, but doing so is complicated by the fact that the bony defect has to be reconstructed. Indeed, the alternative to reconstructing the bony defect is to leave the patient with a craniectomy which places the patient at risk for trauma to the region, as well as wound breakdown over exposed parenchyma given that these regions will be irradiated in the near future. Thus, whenever possible, it is preferable to perform cranioplasty for calvarial-based lesions as soon as possible after surgery. Here, we argue for in situ cranioplasty. 

Given that an in situ cranioplasty is done at the time of tumor resection, it is generally not possible to perform a computer-aided regeneration of the synthetic bone flap. Thus, it is necessary to use techniques to reconstruct the bone flap at the time of surgery, which we describe above. In addition to being a fast method to place the bone flap, the in situ cranioplasty is inexpensive and very durable: the mesh and methyl methacrylate act as steel rebar and cement, providing both compressive and tensile strength to the bone flap. These methods were initially described by Bloch et al. for hyperostosing meningiomas, but are extended in this case to metastatic lesions of the calvarium [5]. 

## Conclusions

The above in situ cranioplasty technique provides a durable and lasting repair of calvarial metastatic lesions, and offers better cosmesis than a free-handed technique.
